# Microbial metacommunity of salt marshes rebuilds along an elevational gradient after initial disturbance

**DOI:** 10.1038/s41598-025-12995-4

**Published:** 2025-07-26

**Authors:** Dennis Alexander Tebbe, Joanne Yong, Mike Smykala, Lucie Kuczynski, Manuel Lanza Guedán, Kertu Lõhmus, Daniela Pieck, Anja Poehlein, Hendrik Schäfer, Martin Könneke, Stefanie D. Moorthi, Bert Engelen

**Affiliations:** 1 Institute for Chemistry and Biology of the Marine Environment, Carl von Ossietzky Universität Oldenburg, Oldenburg, Germany; 2 Institute of Biology and Environmental Sciences, Carl von Ossietzky Universität Oldenburg, Oldenburg, Germany; 3 Institute of Microbiology and Genetics, Georg-August University of Göttingen, Göttingen, Germany; 4 School of Life Sciences, University of Warwick, Coventry, UK

**Keywords:** Salt marsh, 16S rRNA, 18S rRNA, Co-occurrence, Metacommunities, Experimental islands, Microbiology, Environmental sciences, Ocean sciences

## Abstract

Salt marshes are ecologically important ecosystems with dynamic nutrient exchange between land and sea. Their zonation along an elevation gradient supports specific communities exhibiting successional patterns. Previous studies have mainly focused on individual domains, with limited attempts to explore interdependencies of community assemblies across domains. Here, we investigated the co-occurrence of prokaryotes and microeukaryotes in natural salt marsh sediments and experimental islands placed in the adjacent tidal flat. The islands contained originally bare and transplanted plots at three different elevations, corresponding to the typical salt marsh zonation. After initial disturbance by the artificial setting, microbial metacommunities re-assembled along the elevation gradient, showing distinct community compositions comparable to those of the natural salt marsh zones. Interkingdom co-occurrence network analysis revealed sub-communities linked to the natural and artificial settings, with *Woeseiaceae*, *Flavobacteriaceae*, and *Rhodobacteraceae* playing important roles as keystone species. The community assembly was primarily driven by habitat filtering. In conclusion, this study provides insights into the assembly, co-occurrence patterns and recovery of microbial communities in salt marsh sediments. The research highlights the importance of elevation in shaping microbial communities. Understanding these ecological mechanisms is crucial for effective salt marsh protection and conservation facing potential threats like strong disturbances by enhanced storm surges.

## Introduction

Salt marshes are characterized by their exceptional productivity and nutrient exchange between land and sea^1^. They are typically structured by varying inundation frequencies along an elevation gradient, that leads seaward from the upper salt marsh (Upp) via the lower salt marsh (Low) and the pioneer zone (Pio) towards the mudflat area (Mud). Across these salt marsh zones and throughout the seasons, diverse organisms, such as plants, algae, fungi, bacteria, and archaea, display successional patterns in their community structures^2–6^. The zone-specific vegetation plays a dominant role in primary production but also in sediment stability, inhibiting potential erosion by waves and tides^7^. However, the microbial production of extracellular polymeric substance (EPS) by microalgae and phototrophic bacteria (microphytobenthos; MPB) becomes more important for stabilization of salt marsh sediments with their increasing abundances from land to sea^8–10^. The produced organic matter, whether liberated by excretion, grazing, or viral lysis of MPB cells, stimulates growth of heterotrophic bacteria and thus enhances the productivity of the whole ecosystem^11–14^. These communities face potential jeopardy through large-scale disturbances by global change, such as sea-level rise or enhanced storm surges, which could have a lasting impact on inundation frequencies and vegetation patterns^15^. As this unique intertidal habitat plays a crucial role in coastal ecosystems and carbon sequestration^16,17^, understanding fundamental ecological mechanisms, such as community assembly, co-occurrence patterns and recovery, are essential for salt marsh protection.

Previous microbiome studies on salt marshes have predominantly focused on individual domains of life, with limited attempts to explore their interdependencies^18–21^. Yet, it is crucial to recognize that the co-occurrence and underlying interactions between species are as substantial as their general distribution and abundance in understanding community assembly mechanisms^22^. Co-occurrence network analyses have proven to be valuable in identifying taxa that play pivotal roles in ecosystems, facilitating the formulation of hypotheses to explore putative connections between organisms^23^. As an example, investigations focusing on the co-occurrence of bacteria and fungi or vegetation types have revealed strong interdependencies^19–21,24^. Assessing the importance of species within a microbial network involves evaluating their impact on network stability, often measured through centrality metrics^25^. These metrics can be estimated by considering factors such as the shortest path through the network (betweenness^26^) or the frequency of species being passed, indicating their importance in the network when information flows randomly (random walks^27^). However, the most common measure is the number of interactions a species has, known as its degree. Species with the highest centrality within the microbial network are referred to as microbial hubs that disproportionately influence microbial community structures^28^.

This study follows a new approach in identifying potential interactions between prokaryotes and microeukaryotes in salt marsh sediments via co-occurrence network analyses. Furthermore, we analysed the recovery of these communities after an initial disturbance event. The disturbance was introduced by setting up a field experiment on artificial islands, which were installed on the back-barrier tidal flat of Spiekeroog Island (Germany (Fig. S1).) The experimental islands were designed to mimic the three elevations of the natural salt marsh zones (Upp, Low, Pio). One half of these islands was transplanted with lower salt marsh vegetation (Trans), while the other half was filled up with mudflat sediments (Bare). This transfer of material into different elevations of the experimental islands posed the initial disturbance for the benthic microorganisms, disrupting the natural environmental salt marsh gradients. Natural salt marsh sites served as untreated controls (Nat). The overall goal of the experiment was to investigate the mechanisms of spontaneous colonization on dispersal-limited experimental islands in comparison to salt marsh zones with unlimited dispersal^29,30^. While some plants, for example *Salicornia* spp. and *Suaeda maritima*, quickly colonized the initially bare plots^30,31^, their coverage was lower compared to the natural sites and the transplanted plots. In our study, we focused on prokaryotes and microeukaryotes by a combined sequencing approach targeting 16S and 18S rRNA genes, complementing previous investigations on the assembly of plants, animals, fungi, and MPB^5,32–36^. Thereby, we addressed the following hypotheses: (i) Prokaryotic and eukaryotic benthic microbial communities are structured along the land-to-sea transect. (ii) These elevation specific communities assemble within the corresponding zones on the experimental islands. (iii) Transplanted vegetation on the experimental islands supports the zone-specific assembly. (iv) The elevational differences are reflected in distinct community modules within a co-occurring network.

## Results

### Benthic microbial communities assemble along the elevational gradient of natural and artificial salt marshes

**Fig. 1:**
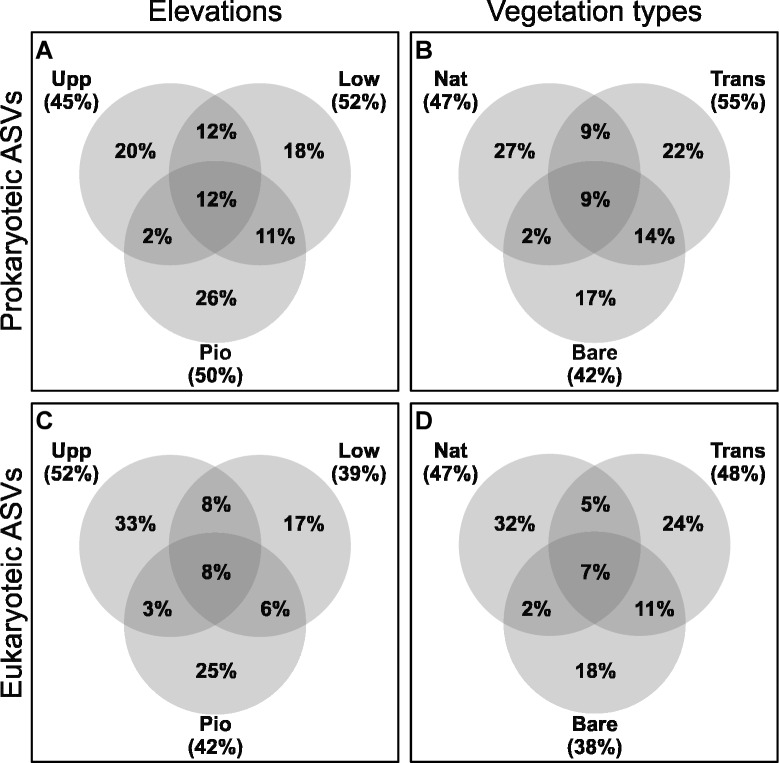
ASVs shared between elevations (Upp, Low, Pio) and vegetation types (Nat, Trans, Bare). The percentage was calculated from a total of 8004 and 3211 unique 16S- and 18S rRNA gene ASVs for prokaryotes (Prok) and eukaryotes (Euk), respectively. Percentages in circles represent the proportions of unique or shared ASVs present in at least one sample (for example in A, 2% of the 8004 ASVs were present in at least one sample from Upp and one sample from Pio). Percentages in brackets are the sums of the ASVs per elevation (**A**,**C**) or vegetation type (**B**,**D**). Samples from the mudflat (Mud) were not included.

**Fig. 2:**
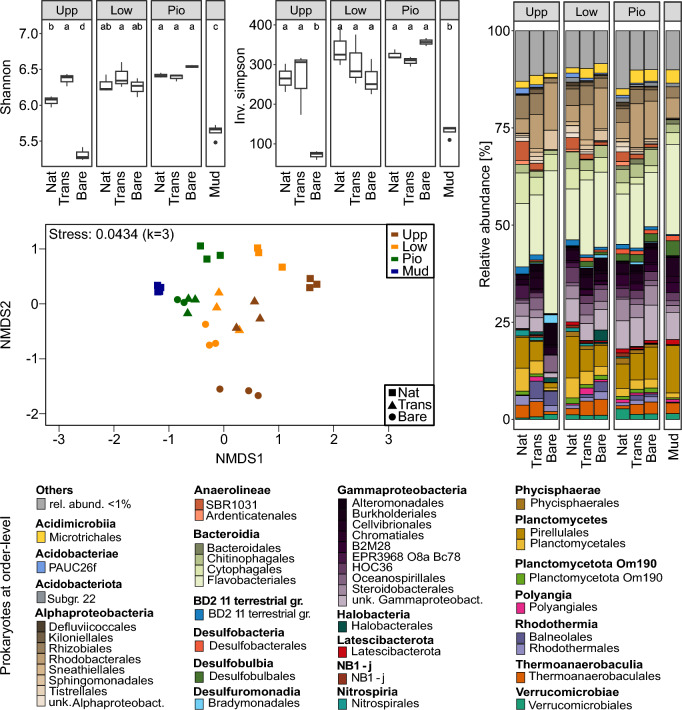
Prokaryotic community assessment. 16S rRNA gene sequencing analysis with alpha diversity measures (Shannon, Inv. Simpson’s), non-metric multidimensional scaling (NMDS), and bar chart of rarefied, normalized mean relative abundances (n = 3).

**Fig. 3:**
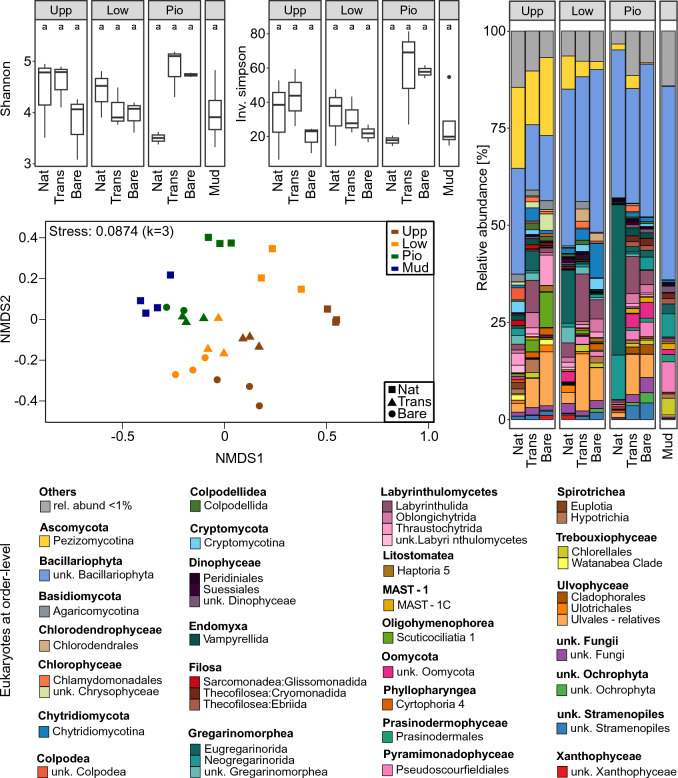
Eukaryotic community assessment. 18S rRNA gene sequencing count data with alpha diversity measures (Shannon, Inv. Simpson’s), non-metric multidimensional scaling (NMDS), and bar chart of rarefied, normalized mean relative abundances (n = 3).

The analysis of shared amplicon sequence variants (ASVs) revealed a general decrease in common ASVs (8004 prokaryotic, 3211 eukaryotic) along with the elevational gradient (Fig. 1). Here, a higher percentage was shared between consecutive elevations, e.g., between the upper salt marsh (Upp) and the lower salt marsh (Low), or Low and the pioneer zone (Pio). Accordingly, the overlap was smaller between the far distant zones (Upp, Pio). The largest fractions were unique to the elevations or vegetation types (17–33%) with smaller percentages of core ASVs (7–12%). While similarities between the artificial sites (Trans, Bare) were overall higher, the vegetated sites (Nat, Trans) had more common ASVs than the natural (Nat) and non-transplanted plots (Bare). Most of these differences between elevations and vegetation types were not reflected in the alpha diversity indices (Figs. 2 and 3). However, substantially lower Shannon and Inv. Simpson’s values were observed for the mudflat (Mud) and some non-transplanted samples (Upp-Bare, Low-Bare).

Consistent shifts in beta diversity (NMDS) were observed for prokaryotic and eukaryotic communities between elevation and vegetation types (Figs. 2 and 3). The analysis demonstrates increased dissimilarities between different elevations of the natural sites (Upp, Low, Pio, Mud). The different settings on the experimental islands showed a similar orientation along the respective elevations, although they were originally filled with mudflat sediment (Bare) or vegetation from the lower salt marsh (Trans). This suggests that the prokaryotic and eukaryotic communities have adapted to the different elevations and partially approached the original community composition on the experimental islands. However, they further divide into patches with higher similarity between the same vegetation types (Nat, Trans, Bare). This shows that the microbial communities still consistently differ from the natural state five years after setting up the experimental islands. The distances between Nat and Trans were lower, compared to Nat and Bare, indicating a seeding or facilitation effect of the transplantation on the microbial community. Since the transplantation experiment was done with material from the lower salt marsh, it was expected that the artificial plots with the corresponding elevation (Trans-Low) would not differ from the natural sites (Nat-Low). Thus, the observed dissimilarity between these samples indicates an incomplete recovery due to the influence of the artificial setup even though the transplanted material was prone to the same environmental conditions including the same inundation frequencies by saltwater.

### Relative abundances of prokaryotic and microeukaryotic communities along salt marsh elevations and vegetation types

The most abundant prokaryotes in the dataset were affiliated to *Bacteroidia*, *Gammaproteobacteria*, *Alphaproteobacteria*, and *Planctomycetes*, comprising means ± standard deviations (SD) of 26% ± 7, 22% ± 5, 16% ± 4, and 10% ± 4 of the total community, respectively (Fig. 2). Some groups, such as *Acidobacteriae*, *Anaerolineae*, *Rhodothermia*, and the BD2-11 terrestrial group, showed decreasing relative abundances along the land-to-sea transect. While many of these groups exhibited higher relative abundances in the natural samples compared to the experimental islands, this was the opposite for *Rhodothermia*. *Desulfobacteria* and *Desulfobulbia* showed higher relative abundances towards the marine sites (Low, Pio) of the experimental islands (Trans, Bare). The *Rhodobacteriaceae* were found in all samples at high relative abundances, with even higher levels in both artificial treatments compared to the natural sites.

Among the eukaryotic groups, *Bacillariophyta* were the most abundant, accounting for 34% ± 14 of the community, followed by *Gregarinomorphea*, *Labyrinthulomycetes*, *Ascomycota*, and *Ulvophyceae*, with relative abundances of 10% ± 16, 8% ± 6, 8% ± 9, and 8% ± 7, respectively (Fig. 3). The relative abundance of *Ascomycota* decreased along the land-to-sea transect, while *Gregarinomorphea* were mainly associated with the natural samples in the lower salt marsh and the pioneer zone (Nat-Low, Nat-Pio). *Labyrinthulomycetes* and *Ulvophyceae* showed higher relative abundances in the artificial plots (Trans, Bare). Within the *Bacillariophyta*, the not further identified Raphid-pennate (21% ± 13) and *Polar*-*centric*-*Mediophyceae* (5% ± 6) were the major representatives, and their relative abundances were lower in the upper salt marsh across all vegetation types.

### Co-occurrence network analysis infers elevation specific community modules within the natural salt marsh and experimental islands

**Fig. 4:**
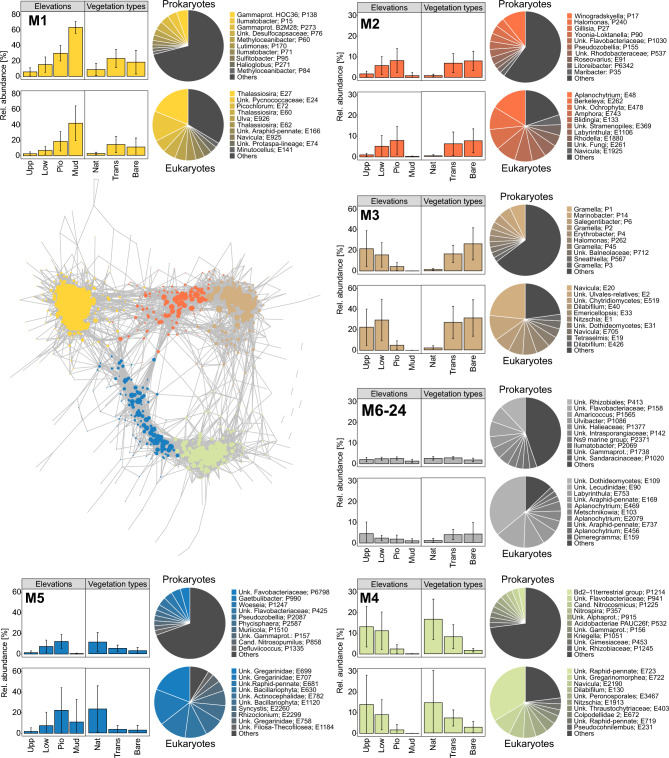
Co-occurrence network analysis. Spearman correlations of prokaryotic 16S rRNA gene ASVs and eukaryotic 18S rRNA gene ASVs (P- and E-numbers). Only genera present in ≥ 25% of the samples were included. Only positive correlations (*r*_*s*_ ≥ 0.7, *p* < 0.01, *q* < 0.05) were considered. The edge distance and node size represent their correlation coefficient (r) and degree of connectivity, respectively. Network modules were identified based on their edge-betweenness. Modules with > 10 nodes were colorized and further characterized in their mean relative module abundances and taxonomic compositions (top 10 most abundant per module), while the remaining grouped (gray).

Microbial co-occurrence network analysis was used to identify co-occurrence patterns and to find potential interactions between and within the prokaryotic and eukaryotic domains (Fig. 4). The network analysis revealed 16,314 positive edges (r_s_ ≥ 0.7, *p* < 0.01, q < 0.05) connecting 799 prokaryotic and 168 eukaryotic nodes, which represent means ± SD of 56% ± 12 and 55% ± 14 of 16S- and 18S rRNA gene reads, respectively (Tab. S2). Based on the edge-betweenness, 24 densely connected community modules were inferred. The five largest modules (> 10 nodes, colored) also contained more than half of the gene reads used for the analysis (Prok: 54%, Euk: 52%). Additionally, 19 small modules were inferred (≤ 10 nodes, gray), each representing minor fractions, only (Prok: 2%, Euk: 3%).

The modules M1–M3 were more abundant in the artificial plots (Trans, Bare) and further separated into the marine module M1, the marine salt marsh module M2 (which increased from land-to-sea with low values in the mudflat) and the terrestrial module M3. The separation between transplanted and non-transplanted plots (Trans, Bare) were not resolved within the experimental island modules M1–M3. Two modules (M4, M5) showed higher relative abundances in the natural sites (Nat) with M4 decreasing from land to the pioneer zone and M5 showing the opposite trend.

Many of the most abundant prokaryotic ASVs within the modules increasing from the upper salt marsh towards the pioneer zone (M2, M5) contained ASVs affiliated to the *Flavobacteriales* (e.g., *Gaetbulibacter*, *Winogradskyella*, *Pseudozobellia*, *Gillisia, Muriicola*). However, these taxa were also part of other modules (e.g., *Gramella* in M3). Another important group was the *Rhodobacterales* (e.g., *Sulfitobacter*, *Roseovarius*, *Litoreibacter*), which was primarily associated with the artificial marine (M2) and terrestrial module M3 mainly from the pioneer zone. *Nitrosopumilus* and candidatus Nitrosocosmicus were the only two *Archaea* found among the most abundant ASVs associated to the natural marine module M5 and the natural terrestrial module M4, respectively.

Diatoms and green algae were within the 10 most relative abundant ASVs of all modules. However, they were most prominent in the marine module M1 with the diatoms *Thalassiosira*, *Navicula*, and *Minutocellus*, and the not further identified Araphid-pennate, as well as the green algae *Pycnococcaceae*, *Picochlorum*, and *Ulva*. Many *Alveolata* were found within the natural salt marsh modules M4and M5. These included e.g., the parasites *Gregarinidae* and *Syncystis* within the marine module M5, as well as the *Colpodellidae* (predatory flagellates) and *Pseudocohnilembus* (ciliates) in the terrestrial module M4. *Stramenophiles*, like *Aplanochytrium*, were found within the most abundant ASVs in the artificial marine module M2 and among the smaller modules (M6-24), while the *Fungi* (*Chytridiomycetes*, *Emericellopsis*, *Dothideomycetes*) showed highly abundant ASVs in the artificial terrestrial module M3.

### Bacterial keystone taxa represent important hubs in network topology

**Fig. 5:**
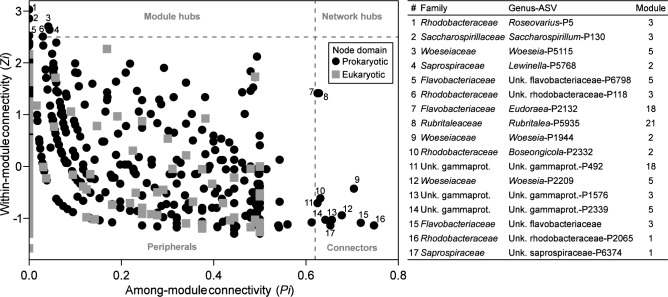
Topological roles of network nodes. Identification of network module hubs within-module connectivity (*Zi*) were plotted over the between network connectivity (*Pi*) to (Zi-Pi plot). According to the simplified network classification^88^, nodes can be categorized into four groups: peripheral nodes (*Zi* ≤ 2.5, *Pi* < 0.62), module hubs (*Zi* > 2.5, *Pi* ≤ 0.62), connectors (*Zi* ≤ 2.5, *Pi* > 0.62), and network hubs (*Zi* > 2.5, *Pi* > 0.62).

In total, 17 prokaryotic but no eukaryotic nodes were identified as important for the network topology with a Zi-Pi analysis (Fig. 5, Table S2). Six of these network hubs (keystone taxa) were characterized as module hubs (*Zi* > 2.5, *Pi* ≤ 0.62) and eleven as connectors (*Zi* ≤ 2.5, *Pi* > 0.62). *Rhodobacteraceae*, *Flavobacteriaceae*, *Woeseiaceae*, and *Saprospiraceae* were groups that contained both, module hub and connector ASVs. Another module hub was within the *Saccharospirillaceae*, while further connectors were affiliated to unknown *Gammaproteobacteria* or *Rubritaleaceae*. *Rhodobacteraceae* stood out as they contributed two connectors and two module hubs, which marks them as an especially important prokaryotic component within the ecosystem. These were all within the artificial modules M1–M3.

### Microbial community dissimilarity increased with differences in elevation but was not clearly affected by geographical distance

**Fig. 6:**
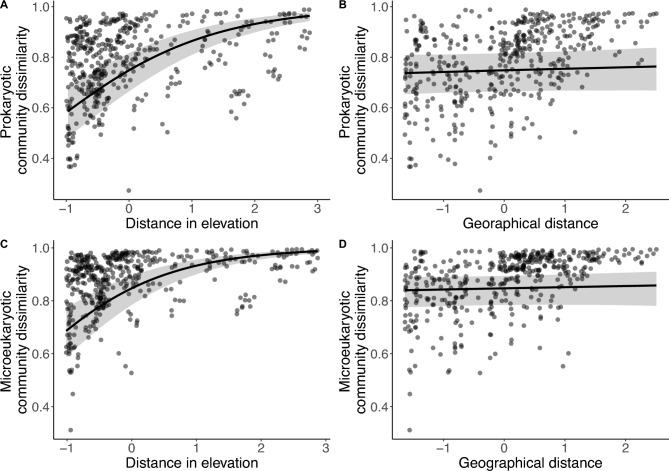
Correlation between community dissimilarities based on Bray–Curtis distances. Left: community dissimilaritiy of prokaryotes (**A**) and microeukaryotes (**C**) plotted over standardized distance in elevation. Right: community dissimilaritiy of prokaryotes (**B**) and microeukaryotes (**D**) plotted over standardized geographical distance.

Both eukaryotes and prokaryotes showed a strong spatial structure in diversity, as the community dissimilarity (Bray–Curtis) was on average high, showing a mean ± SD for prokaryotes of 0.80 ± 0.17 and 0.82 ± 0.17 for eukaryotes. Community dissimilarity in both prokaryotes and eukaryotes was more strongly related to differences in elevation than to geographical distance (Fig. 6). For prokaryotic communities, greater differences in elevation between sites were associated with higher dissimilarity (posterior estimate = 0.76, 95% credible interval: 0.67–0.85), while geographical distance showed no meaningful effect (estimate = 0.03, 95% CI − 0.05 to 0.11). Similarly, eukaryotic community dissimilarity increased with difference in elevation (estimate = 0.92, 95% CI 0.81–1.03), but not with geographical distance (estimate = 0.03, 95% CI − 0.06 to 0.13). The models also captured considerable variability in baseline dissimilarity between sites (site-level SDs: 0.74–0.84), and the estimated precision parameters (ϕ) indicated low residual variance for both models (ϕ_proks = 41.82; ϕ_euks = 46.10), suggesting that elevation is a strong predictor of community dissimilarity. Therefore, the relationship between the elevational gradient and community dissimilarity suggests habitat filtering to play a key role for community assembly.

## Discussion

In this study, we focused on the relationships within and between prokaryotic and microeukaryotic communities from salt marsh sediments. These benthic microorganisms are an integral part determining salt marsh ecology, and their community composition is influenced by a variety of biotic interactions. Our analyses confirm previous observations that prokaryotic and microeukaryotic community compositions shift along a land-to-sea gradient^2,36–38^. We also observed this land to sea shift on the investigated experimental islands, even though there was a strong influence by the experimental setup. With the co-occurrence network analysis, we were able to infer the respective sub-communities associated to the natural (Nat) salt marsh zones (Upp, Low, Pio, Mud) or to one of the conditions on the experimental islands (Trans, Bare). In addition to the identification of these community modules and the NMDS analysis, we observed that the community assembly appears to be dispersal limited.

While most previous ecological studies on salt marshes analyzed community structures and differences within single domains^20^, recent investigations on coastal wetlands focused more on the interaction between the domains of life^39–41^. In our study, we followed this approach and hypothesized that prokaryotic and eukaryotic communities would be structured along the natural land-to-sea gradient from the upper salt marsh to the mudflat. The observed shifts in beta diversity (Fig. 2) changing with factors like inundation frequencies and distance to sea, support our hypothesis and are in accordance with previous studies on the zonation of bacterial salt marsh communities^2,37,38,42^. The shifting relative abundances from land to sea were evident, as seen for example in the decrease of the typical soil bacteria *Acidobacteriae*^43^ and the increase of the sulfate reducing *Desulfobulbales*, typically found in intertidal sediments^44^. On the other hand, the metabolically versatile *Rhodobacteriaceae* and *Flavobacteriales* were found in all samples with higher relative abundances in the marine plots^45^. These heterotrophs complete the nutrient cycle by remineralizing organic matter which is produced by green algae and diatoms and returning essential elements back to those primary producers (e.g. *Thalassiosira*, *Navicula*, and *Minutocellus*)^46^. Both groups of prokaryotes and eukaryotes were found in the same marine modules (M1, M2 and M5). Besides this trophic interaction, there are more complex ecological relationships between and within prokaryotes and microbial eukaryotes affecting microbial community structures.

Here, we could provide further evidence that the observed shift in beta diversity also applies to a broad range of microeukaryotes (Fig. 3). While diatoms (*Bacillariophyta*) were ubiquitous and prevailed in the more marine sites, *Fungi* (e.g., *Ascomycota*) were strongly associated to the terrestrial sites. In coastal marine sediments, the interactions in benthic microbial food webs are highly complex, including competitive, symbiotic and feeding interactions among and within prokaryotes and microbial eukaryotes that determine community structures^47^. The feeding patterns of protists can be very selective, and thereby shape algal and bacterial communities^48,49^. For instance, we observed increasing relative abundances of *Gregarinomorphea* towards the pioneer zone. Among those, the *Eugregarinorida* are found in marine and terrestrial habitats, some in symbiosis with mollusks and crustaceans, whereas *Neogregarinorida* are common in terrestrial hosts like insects^50^. The *Labyrinthulomycetes*, were present in all salt marsh samples, with increased relative abundances on the experimental islands. Their importance as decomposers in other highly productive intertidal systems, like mangroves and seagrass meadows^51,52^, indicates that they probably are important organic matter degradation in salt marshes.

The higher similarities between vegetated sites (Nat, Trans) also emphasize the pivotal role of vegetation in the assembly of prokaryotic and microeukaryotic communities in salt marsh sediments. Our observations generally align with previous studies on other salt marshes that have shown a strong influence of plant community composition on bacterial community structures^53^ and the restoration of MPB communities^54^. For example, an enrichment of plant root-associated microorganisms has been observed across many environments and plant species^41,55,56^. Additionally, the roots increase sedimentation of fine materials, which retains the moisture in the sediments^57^. To certain extent, vegetation is also beneficial as the leaf shade protects the benthos from temperature peaks during days of high solar radiation and desiccation^57^. Furthermore, plant phenotype and the migration of plant species can substantially impact bacterial and fungal communities^58–60^.

The experimental islands were set up to investigate whether salt marsh communities would develop according to the natural zonation, or if dispersal limitation would prevent this assembly^30^. Although all Bare plots were filled with mudflat sediments and Trans plots with material transplanted from the lower salt marsh, our NMDS analysis shows a clear shift towards elevation specific community compositions. In contrast, there must have been an overarching effect of the artificial setup, indicated by the divergence of the microbial communities from the lower salt marsh (Low-Nat) transplanted to the corresponding elevation on the experimental island (Low-Trans). If elevation was the only factor for community assembly, the transplanted lower salt marsh would still be composed of the same community members. While this environment is prone to a delicate balance between formation and erosion^61^, the community assembly appears to be resilient^62^. The described development of elevation associated communities on both, bare and transplanted artificial islands, emphasize that environmental forces specific to particular elevations strongly determine microbial community composition five years after the initial disturbance. However, they have not fully recovered yet and still differed from the natural habitat.

With the co-occurrence network analysis, we were able to identify sub-communities (modules) reflecting the elevational gradients and the effects of the artificial setup. These analyses offer a valuable approach to confirm or detect potential interactions in nature^23^. We identified modules associated to the artificial plots (M1–M3) and the natural sites (M4, M5), which further divide into marine (M1, M2, M5) and terrestrial sub-communities (M3, M4). Our Zi–Pi-analysis showed that the *Woeseiaceae*, *Flavobacteriaceae*, and *Rhodobacteraceae* are keystone taxa across the salt marsh environment, important for module- and network stability as both, module hubs and connectors. However, detailed investigations of single interaction e.g., in co-culture experiments are important to understand specific interactions, which usually require simplifications to draw meaningful conclusions and thereby exclude the environmental complexity^63^.

To explain the assembly of communities, generally, two prevailing theories with contrasting perspectives are used. While the niche-based theory assumes non-random, deterministic dynamics (e.g., mutualism, facilitation, competition, selection or predation), the neutral theory proposes that communities assemble randomly, governed by stochastic processes such as dispersal limitation, ecological drift, and random speciation^18,64–66^. In our study, the Bayesian regression model indicate that the community turnover is strongly shaped by habitat filtering (i.e. elevational gradient), whereas dispersal limitation (i.e. geographic distance) shows a subordinate role. Thus, our results support the niche-based theory, showing habitat filtering along the elevational gradient for both prokaryotes and microeukaryotes.. A similar assembly mechanism was found for bacterial communities within salt marsh sediments of Hangzhou Bay (China) which have been shown to progress from stochastic to deterministic with increasing terrestrial influence^37^. While the underlying mechanisms of the niche-based and neutral theories interact and occur simultaneously, the extent and importance of these deterministic and stochastic effects are still under debate^67,68^.

## Concluding remarks

We showed that prokaryotes and microeukaryotes in salt marshes are forming distinctive sub-communities that assemble along a transect from land-to-sea by habitat filtering. The network analysis revealed deeper insights into the co-occurrence of both domains. The identified modules clearly separated the natural salt marshes and the artificial settings, but both assembled along the elevational gradient. This association was restored even from bare mudflat sediments when facing the same inundation frequencies. Event though the microbial communities did not fully recover, their ability to rebuild along the elevational gradient indicates their capability to respond to disturbances.

## Materials and methods

### Sampling

Samplings were performed on Spiekeroog Island, North Sea, Germany, on the 16th of July 2019 in the frame of the DynaCom project (“Spatial community ecology in highly dynamic landscapes: from island biogeography to metaecosystems”, FOR2716). Samples were taken along three land-to-sea transects in the upper salt marsh zone (Upp), lower salt marsh zone (Low) and pioneer zone (Pio) within the natural salt marsh (Nat) and on 6 experimental islands, located on the backbarrier tidal flat of Spiekeroog island (Fig. S1, Table S1). The detailed description of the experimental islands which were set up five years prior to the sampling is given in Balke, et al.^30^ . In short, a total of 12 experimental islands were positioned at the same elevation of ~ 80 cm (related to standard elevation zero, NHN), 60–120 m apart from each other and a distance of 240–460 m to shore. They are made of 2 × 6 m large steel cages, containing 4 smaller cages (100 × 100 cm) per elevational level, representing the 3 different zones of the salt marsh (Pio = 70 cm, Low = 100 cm, Upp = 130 cm NHN) and experience the same inundation frequencies as the corresponding natural zones. Six islands were filled up to 10 cm below the top of each cage to create bare islands for an initial colonization experiment (Bare). For a transplantation experiment, six islands were additionally planted with sods from the lower salt marsh on top of the tidal-flat sediment (Trans). For the present study, the islands 7, 9 and 11 (Trans) and 8, 10, 12 (Bare) were sampled. Geo locations (GPS), elevations (altitude), and abiotic factors were measured for the sampling sites as described before^29,69^. In addition, four sites were sampled in the mudflat (Mud), which were 200 m apart, each. For all sites, triplicates of 1 cm^3^ sediment from the top 0–1 cm were collected with sterile cut-off syringes and frozen in the field. To obtain more representative bulk sediment samples, the replicates from each sampling site were mixed prior to DNA extraction.

### DNA extraction and sequencing

DNA extraction was performed using 0.5 g (wet weight) of sediment and the DNeasy PowerSoil Pro Kit (Qiagen, Hilden, Germany) following the manufacturer’s instructions. DNA was resuspended in PCR-grade water and stored at – 20 °C after quantification and quality assessment using a NanoDrop spectrophotometer (Thermo Fisher Scientific, Bremen, Germany). To amplify the hypervariable regions V4–V5 of the 16S rRNA gene, the 515F-Y (5′-GTG YCA GCM GCC GCG GTA A-3′) and 926R (5′-CCG YCA ATT YMT TTR AGT TT-3′) primers were used^70^. Amplification and library preparation procedures were conducted as described by Yeh, et al.^71^. For 18S rRNA gene sequencing, the V4 region was amplified using the V4 18S Next.For (5′-CCA GCA SCY GCG GTA ATT CC-3′) and V4 18S Next.Rev (5′-ACT TTC GTT CTT GAT YRA TGA-3′) primers^72^. Amplification and library preparation was done as described by Piredda, et al.^72^.

### Processing of amplicon sequences

The 16S rRNA and 18S rRNA gene reads were processed using qiime2-2021.2^73^, including the denoising algorithm DADA2, further described in the pipeline of Yeh, et al.^74^. To ensure reproducibility, a standardized conda environment was employed. The scripts utilized in this analysis were adapted from the collection available at: https://github.com/jcmcnch/eASV-pipeline-for-515Y-926R. In summary, the following steps were performed: Primer removal and trimming were carried out using the cutadapt package^75^, allowing a 20% primer mismatch. The 16S rRNA or 18S rRNA datasets were then selected using the bbtools package and SILVA138^76^ or Protist Ribosomal Reference (PR^2^) database version 4.14.0^77^, respectively. Low-quality ends (Quality score < 30) were removed by trimming the forward and reverse sequences at 230 bp and 210 bp for 16S rRNA gene sequences, and at 220 bp and 212 bp for 18S rRNA gene sequences. Denoising, merging, and chimeric removal were performed using the qiime2 dada2 plugin^78^. Taxonomic classification of amplicon sequence variants (ASVs) was accomplished using the qiime2 classify-sklearn plugin with SILVA138 or Protist Ribosomal Reference (PR2) database version 4.14.0 as the reference databases. Sequences assigned to chloroplasts and mitochondria were excluded from all subsequent analyses.

### Prokaryotic and microeukaryotic diversity

All statistical analyses were conducted using R^79^. Alpha diversity measures, rarefication, non-metric multidimensional scaling (NMDS), and Bray–Curtis dissimilarities were determined using the vegan 2.6-4 package^80^. Read counts for shared ASV, alpha-, beta diversity and co-occurrence network analyses were rarefied to the samples with the minimum number of counts for 16S rRNA sequences (13,602) and 18S rRNA sequences (12,627). To minimize sub-sampling randomness, rare counts were averaged from repeated rarefication (999x). The focus of the study was on microorganisms, thus the unknown *Eukaryota*, *Streptophyta*, *Metazoa*, and unknown *Opisthokonta* from the 18S rRNA gene data were removed after rarefication, as these groups mainly contain multicellular organisms or do not provide valuable information.

Venn diagrams illustrating shared ASVs were generated using the VennDiagram 1.7.3 package^81^. Statistical differences in alpha-diversity indices were assessed with the analysis of variance (ANOVA) from the aov function from stats package 4.3.0^79^ and Tukey’s Honest Significant Difference (Tukey’s HSD) test with agricolae 1.3-5^82^, considering *p* < 0.01 (ANOVA) and alpha < 0.05 as significantly different. Bar plots depicting relative abundances were organized at the order level, highlighting taxa with relative abundances exceeding 1% in at least 10% of the samples, while the remaining taxa were grouped as “Others”. For NMDS analysis, Bray–Curtis dissimilarities were calculated based on log-transformed proportions to mitigate the asymmetry of ASV distributions. Statistical significance between groups was evaluated using permutational multivariate analysis of variance (PERMANOVA) with the pairwiseAdonis 0.4 package^83^.

### Network construction and characterization

Co-occurrence network inference was done based on Spearman-correlation matrix and Benjamini–Hochberg adjusted p-values using the corr.test function from the psych 2.2.9 package^84^. A combined feature list of 16S- and 18S rRNA ASVs present in ≥ 25% of samples were used for the correlation. The network analysis was done with the igraph 1.4.1 package^85^ where each dot (Node) represents a different ASV. Only strongly positive correlations were considered (r_s_ ≥ 0.7, *p* < 0.01, q < 0.05), applying r_s_ (Spearman’s rank correlation coefficient) as a measure of the strength and direction of the monotonic relationship between two variables, the statistical significance *p* and the multiple testing correction q, accounting for multiple comparisons using the false discovery rate (FDR). For graphic presentation (Fig. 4), positive correlations are displayed as lines between the nodes. The edge distance represent their correlation coefficient (r) and the node sizes the number of connections i.e., degree of connectivity. Community modules were calculated based on the edge-betweenness (cluster_edge_betweenness) and thus reflects densely connected nodes (modules), which are sparsely connected to other modules. Modules with more than ten nodes were colorized and further characterized in their mean relative module abundances (bar charts) and taxonomic compositions (pie charts).

Topological roles of individual nodes were described using the within-module connectivity (*Zi*) and the between network connectivity (*Pi*)^28,86^. Node degrees and community modules from the network analysis described above were used to calculate the *Zi* and *Pi* (amended R script from Cao, et al.^87^). Nodes can be classified into four groups using a Zi-Pi analysis^88^: peripheral nodes (*Zi* ≤ 2.5, *Pi* < 0.62), module hubs (*Zi* > 2.5, *Pi* ≤ 0.62), connectors (*Zi* ≤ 2.5, *Pi* > 0.62), and network hubs (*Zi* > 2.5, *Pi* > 0.62).

### Assessment of community assembly

Beta diversity between communities for the prokaryotes and eukaryotes were quantified separately as the Bray–Curtis dissimilarity to infer community assembly. To evaluate how environmental and geographical distances (Table S1) shape microbial community dissimilarity, we employed a Bayesian regression model using the brms package in R^89^. Pairwise dissimilarity of microbial communities was modeled as a function of standardized (z-transformed) differences in altitude and geographical distance between sampling sites. Due to the bounded nature of Bray–Curtis dissimilarities (0 < y < 1), we used a Beta distribution with a logit link function. Random intercepts were included for both sites in each site pair to account for repeated measures and spatial autocorrelation. The model was fit using the brm() function, running 4 Markov chains with 9999 iterations each (4999 warm-up), utilizing the No-U-Turn Sampler (NUTS) via Stan. We confirmed model convergence (R̂ < 1.01) and performed posterior predictive checks to assess fit. All continuous predictors were standardized (z-transformed), and results are reported as posterior means with 95% credible intervals.

## Supplementary Information

Supplementary Information.

## Data Availability

The raw reads of 16S rRNA and 18S rRNA amplicon sequencing were deposited in the European Nucleotide Archive (ENA) at EMBL-EBI under accession number PRJEB66381 (https://www.ebi.ac.uk/).
