# Supplementary material for: Microbial metacommunity of salt marshes rebuilds along an elevational gradient after initial disturbance
Source: Sci Rep. 2025 Jul 26;15:27178. doi: 10.1038/s41598-025-12995-4 (PMC12297458; doi:10.1038/s41598-025-12995-4)
Supplement: Supplementary file 1 — Supplementary Information. [file 41598_2025_12995_MOESM1_ESM.pdf]

Microbial metacommunity of salt marshes rebuilds along an elevational gradient after initial disturbance - Supplemental material

Dennis Alexander Tebbe[1], Joanne Yong[1], Mike Smykala[1], Lucie Kuczynski[1], Manuel Lanza Guedán[2], Kertu Lõhmus[2], Daniela Pieck[1], Anja Poehlein[3], Hendrik Schäfer[4], Martin Könneke[1], Stefanie D. Moorthi[1], Bert Engelen[1*]

[1]Carl von Ossietzky Universität Oldenburg, Institute for Chemistry and Biology of the Marine Environment, Oldenburg, Germany

[2]Carl von Ossietzky Universität Oldenburg, Institute of Biology and Environmental Sciences, Oldenburg, Germany

[3]Georg-August University of Göttingen, Institute of Microbiology and Genetics, Göttingen, Germany

[4]University of Warwick, School of Life Sciences, Coventry, United Kingdom

*engelen@icbm.de

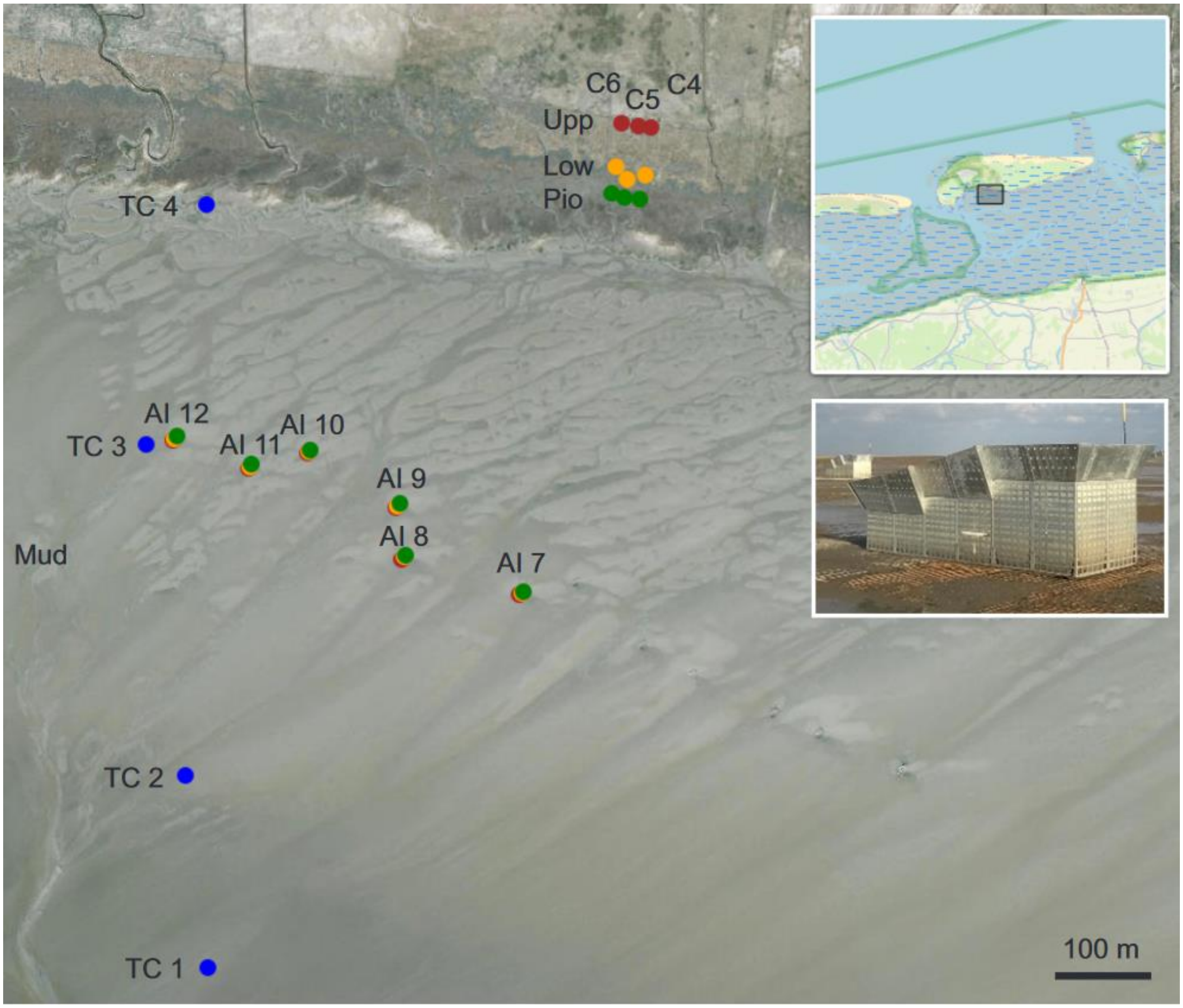

Figure S1: Map of the sampling sites located at the southern side of Spiekeroog island, Germany. Colors represent sampling sites in the upper salt marsh (red), lower salt marsh (orange), pioneer zone (green), and mudflat (blue). Experimental islands were setup in the intertidal mudflat area, represented by the same color code as for the different salt marsh zones.

Table S1: Sample table including categorical information (Setup, Elevation, Condition), GPS data (Altitude, Latitude, Longitude), and some abiotic sediment parameter: Temperature (Temp. °C), water content (H_2O), Density ($g \cdot cm^{-3}$), and oxygen at different sediment depths.

Sample_ID	Setup	Elevation	Condition	Altitude	Latitude	Longitude	Temp.	H_2O (%)	Density	O_2 0cm	O_2 0.5cm	O_2 1cm	O_2 1.5cm	O_2 2cm
SP-Upp-C4	SP	Upp	Nat	1.88270	53.76262	7.72290	NA	NA	NA	NA	NA	NA	NA	NA
SP-Upp-C5	SP	Upp	Nat	1.83750	53.76264	7.72270	NA	NA	NA	NA	NA	NA	NA	NA
SP-Upp-C6	SP	Upp	Nat	1.80630	53.76267	7.72245	NA	NA	NA	NA	NA	NA	NA	NA
SP-Low-C4	SP	Low	Nat	1.53620	53.76220	7.72280	15.3	57.74	0.60	276.5	289.6	291.5	300.9	313.2
SP-Low-C5	SP	Low	Nat	1.51245	53.76216	7.72252	NA	NA	NA	NA	NA	NA	NA	NA
SP-Low-C6	SP	Low	Nat	1.69240	53.76227	7.72236	16.3	48.36	0.71	274.6	279.8	309.7	302.8	306
SP-Pio-C4	SP	Pio	Nat	1.26900	53.76198	7.72272	16.8	60.56	0.62	266.1	260.9	254.3	249.5	242.9
SP-Pio-C5	SP	Pio	Nat	1.32255	53.76200	7.72250	NA	NA	NA	NA	NA	NA	NA	NA
SP-Pio-C6	SP	Pio	Nat	1.35910	53.76203	7.72229	16.2	56.94	0.62	267.3	180.8	167.2	175.6	149.3
Mud-Mud-TC 4	Mud	Mud	Mud	0.85380	53.76193	7.71609	18	34.45	2.38	264.8	254.2	254	223.8	219.5
Mud-Mud-TC 3	Mud	Mud	Mud	0.58120	53.75977	7.71516	18.1	18.77	1.48	236.1	252.8	254.4	253.5	251.4
Mud-Mud-TC 2	Mud	Mud	Mud	0.06620	53.75677	7.71577	17.6	18.25	0.92	267.1	241.2	186.3	161.7	144.9
Mud-Mud-TC 1	Mud	Mud	Mud	0.02550	53.75503	7.71611	17.9	31.10	2.32	229.7	223	196.3	188.3	182.8
AI-Upp-7	AI	Upp	Trans	2.00710	53.75841	7.72089	14.6	30.55	1.13	280.1	281	284.5	285.7	283.1
AI-Upp-9	AI	Upp	Trans	1.96730	53.75920	7.71900	16.7	19.86	1.53	270.8	270.7	276.3	282.6	282.4
AI-Upp-11	AI	Upp	Trans	1.98590	53.75955	7.71673	18	30.05	1.84	267.4	276.1	273.4	277.3	278.6
AI-Low-7	AI	Low	Trans	1.76410	53.75842	7.72092	14.9	24.68	1.50	278.8	287.7	289.2	289.2	280
AI-Low-9	AI	Low	Trans	1.74130	53.75921	7.71902	17.2	21.49	1.04	265.7	269.9	272.7	278.9	284.4
AI-Low-11	AI	Low	Trans	1.75200	53.75956	7.71676	17.8	20.04	1.28	272.7	286.1	285.7	281.2	256.2
AI-Pio-7	AI	Pio	Trans	1.47620	53.75843	7.72094	15.1	26.03	1.99	273.6	293.9	294.4	290.1	291.9
AI-Pio-9	AI	Pio	Trans	1.50830	53.75922	7.71906	16.8	22.72	2.02	267.9	288.1	282.1	278.7	276.1
AI-Pio-11	AI	Pio	Trans	1.44000	53.75958	7.71678	17	29.72	2.35	264.1	250.4	246.6	223	204.4
AI-Upp-8	AI	Upp	Bare	1.84670	53.75873	7.71908	15.8	6.92	1.29	273	272.5	273.6	274.6	274.8
AI-Upp-10	AI	Upp	Bare	2.00300	53.75969	7.71763	19	14.55	2.57	244.7	252	254.4	255.4	256.7
AI-Upp-12	AI	Upp	Bare	1.97940	53.75980	7.71559	22	8.56	1.98	251.1	254.2	255.5	256.6	256.1
AI-Low-8	AI	Low	Bare	1.68770	53.75874	7.71912	15.3	16.22	1.03	274.4	276.7	276.9	277.9	278.7
AI-Low-10	AI	Low	Bare	1.71400	53.75970	7.71766	16.6	19.75	1.80	235.8	237.1	239.7	254.1	250
AI-Low-12	AI	Low	Bare	1.71240	53.75982	7.71561	22.7	12.90	0.72	236.2	243	244.6	246	247.6
AI-Pio-8	AI	Pio	Bare	1.37970	53.75875	7.71915	14.9	29.24	1.70	280.8	308.4	305.8	292	290.8
AI-Pio-10	AI	Pio	Bare	1.41200	53.75972	7.71769	16.5	26.83	2.42	267.6	278.7	280.2	276.9	272.7
AI-Pio-12	AI	Pio	Bare	1.41440	53.75984	7.71565	18.6	33.68	4.31	260.4	238.8	183.7	173.8	168.4

Table S2: Community module statistics. Relative abundance of the prokaryotic (Prok abun.), eukaryotic (Euk abun.), and combined abundance per module (Total abun.). The number of nodes of the prokaryotic (Prok nodes), eukaryotic (Euk nodes), and combined nodes per module (Total nodes).

Module	Euk abun.	Prok abun.	Total abun.	Prok nodes	Euk nodes	Total nodes
1	13.0	22.0	35.0	264	55	319
2	4.4	5.0	9.4	93	20	113
3	17.1	12.5	29.5	151	25	176
4	7.3	8.0	15.4	130	28	158
5	10.2	6.2	16.3	114	19	133
6	0.0	0.2	0.2	3	0	3
7	0.0	0.1	0.1	2	0	2
8	0.0	0.0	0.0	2	0	2
9	0.0	0.1	0.1	2	0	2
10	0.0	0.0	0.0	2	0	2
11	0.0	0.4	0.4	7	0	7
12	0.1	0.4	0.6	6	1	7
13	0.7	0.0	0.7	1	8	9
14	0.0	0.2	0.2	6	1	7
15	0.3	0.3	0.6	3	2	5
16	0.2	0.0	0.2	1	1	2
17	1.0	0.0	1.0	1	1	2
18	0.0	0.2	0.2	3	0	3
19	0.0	0.0	0.0	2	0	2
20	0.0	0.1	0.1	2	0	2
21	0.0	0.1	0.1	2	1	3
22	0.0	0.1	0.1	2	0	2
23	0.3	0.0	0.3	0	4	4
24	0.1	0.0	0.1	0	2	2
